# Which competencies should be fostered in education for sustainable development at higher education institutions? Findings from the evaluation of the study programs at the University of Bern, Switzerland

**DOI:** 10.1007/s43621-023-00134-w

**Published:** 2023-03-28

**Authors:** Thomas Hammer, Anna Lena Lewis

**Affiliations:** Centre for Development and Environment (CDE), University of Bern, Mittelstrasse 43, 3012 Bern, Switzerland

**Keywords:** Sustainability competencies in higher education, Curricula development, Evaluation of study programs, University of Bern/Switzerland

## Abstract

A relatively broad consolidated consensus has emerged among experts regarding the competencies that should be fostered through an education for sustainable development at the higher education level. However, there is little empirical support to aid in answering the question of which competencies should be promoted from the perspective of students and graduates. This was the main purpose for analyzing the corresponding results of the evaluation of the study programs in sustainable development at the University of Bern. In a standardized survey, students (N = 124), graduates (N = 121), and the supervisors of internships (N = 37) were asked, among other questions, how important they consider the fostering of the respective 13 competencies during their studies and for their professional activities. Overall, the results confirm the view of experts: the study programs should be designed for a comprehensive empowerment with respect of responsible and self-motivated participation in meeting the challenges of sustainable development. Even the students are of the opinion that competency-oriented education is important and that not only the acquisition, respectively the imparting of knowledge is relevant. Regarding the estimation of the promotion of competencies in the study program, the three groups agree that the competencies “Interconnected, foresighted, and thinking approaches in system-dynamic contexts” and “Recognizing on one’s own perspective on a situation and problem, empathizing with other perspectives, and taking these into account when solving problems” are the most important. For the professional field, the competency “Communicating in a comprehensive and target group-oriented manner” is rated most important by all three groups. However, it must be noted that there are also differences between the varying perspectives of the students, graduates, and internship supervisors. The results indicate opportunities for improvement that can also be considered as recommendations in the further development of inter- and transdisciplinary sustainability-oriented study programs. Furthermore, lecturers should, especially regarding a multidisciplinary team, coordinate and communize the development of competencies across the different educational elements. Students should be well informed regarding how the various educational elements, i.e., teaching/learning arrangements and assessments, are intended to contribute to the overall development of competency. Finally, in order to ensure that lecturers align respective learning outcomes, as well as teaching/learning arrangements and assessments in their educational elements, there will need to be a greater focus on competency development across a program of study.

## Introduction

The greatest challenge at the present period is to conserve the planet's resources, while also developing welfare for all and to effectively maintain a growing population. As the urgency and speed of global change increase, so too does societies’ need for knowledge and solutions in order to meet these challenges [1]. Education is to be assigned a key role in demanding that it contributes to progress toward a sustainable society [2]. Riess et al. [3] use the term “Education for Sustainable Development” (ESD) to describe the “totality of all actions by which people seek to promote the competencies of learners in such a way as to enable them to shape sustainable development” [3, p. 298]. Through holistic, inter-, and transdisciplinary learning methods, ESD should sensitize people and enable them to shape future developments in a responsible way and thus render innovative contributions to all economic, social, ecological, and cultural topics—thus resulting in a positive contribution to society [4, 5].

Higher education institutions as engines of research and innovation for the cultural, socio-economic, and ecologically sustainable development of individuals, communities, and nations are of major importance in regard to this aim [1]. Sustainability-oriented study programs render contributions at various levels, that is: They ensure that knowledge on the ecological and social consequences of modern societies, as well as in regard to suitable strategies for the purposes of dealing with these challenges are translated into courses. Transdisciplinary courses and multi-stakeholder approaches introduce students to new forms of thinking, acting, and research. As such, they ensure the education and sensitization of future sustainability scientists.

In respect to the discussion on Education for Sustainable Development (ESD), a broad consensus has emerged that education in respect of sustainable development should also be designed in a competency-oriented framework at the higher education level [6–10]. Competencies should represent the overarching orientation framework for curriculum development and all associated pedagogical-didactic requirements in respect of teaching (which includes lecturer training, as well as the development of teaching modules, including the derivation and the justification of learning outcomes, teaching–learning arrangements, and assessments) [11–15]. Likewise, a certain consensus has evolved in respect of the fact that competency development should be understood more broadly than the acquisition of knowledge, skills, and attitudes (often referred to as “instrumental” ESD). Instead, students should be enabled to deal critically, reflexively, and inclusively with various values, concepts, and solution approaches [16–18] and thus contribute responsibly to overcoming real-world problems, opportunities, and challenges in their spheres of activity and action (i.e., the so-called “emancipatory” ESD) [19]. According to Wiek et al. [20, p. 204], competencies consist of “knowledge, skills and attitudes that enable successful task performance and problem solving with respect to real-world sustainability problems, challenges and opportunities”. Competencies can also be considered as integrated skills, consisting of an interplay of knowledge, skills, and attitudes that enable people to assume responsibility and to participate in problem-solving processes in a targeted and effective manner at work, in an organization, in civic engagement, and in everyday life [21]. Moreover, according to Glasser and Hirsh [22, p. 126], competencies consist of a “constellation of abilities, attitudes, knowledge, understanding, skills, and habits of mind that are functionally linked to support both problem-posing and problem-solving and evoke purposeful behavior toward particular end goals.”

Ever since the Bologna reform in higher education, specifically in the context of ESD, various competency frameworks have been conceptualized across educational levels or specifically developed for the purposes of the higher education level. An overview is provided by Rieckmann [23]; Riess et al. [3]; as well as Wilhelm et al. [15]. In addition, we must also include the competency framework of de Haan [24–26] and Bormann and de Haan [27], which has entered the literature under the terms “Gestaltungskompetenz”, and “shaping competence”, respectively. Furthermore, the competency frameworks of Lozano et al. [11]; Rieckmann [28, 29]; Glasser and Hirsh [22]; Cebrián and Junyent [30]; Dlouhá et al. [31]; Wals [16, 32]; Wiek et al. [20]; as well as that of Wiek et al. [33]. The latter has been further developed by Brundiers et al. [7] by means of a Delphi study and is in our opinion the actual and most appropriate reference framework at this time.

Shephard et al. [34] also identified the differences and contradictions between the various conceptions of competencies. However, in the sense of Rieckmann [23], there is some consensus on what the key sustainability competencies are. He includes the following competencies, which are included in certain competency frameworks: systems-thinking competency; anticipatory competency; normative competency; strategic competency; collaboration competency; critical thinking competency; self-awareness competency; and integrated problem-solving competency—whereby the latter, in respect of the interaction with the other competencies, is to be understood as a framework competency that enables one to contribute to the mastering of sustainability problems [see also 9].

The various competency frameworks have been developed theoretically, for example, in the frame of Delphi surveys of researchers and lecturers [7, 28, 29] or in the frame of surveys of professionals [35–37]. However, there are hardly any empirical studies on which competencies students and graduates of sustainability-oriented study programs consider important. Exceptions include the studies by Brudermann et al. [38] (in their survey of students at the University of Graz/Austria) and Hammer and Pfäffli [39] (in their survey of students at the University of Bern/Switzerland). This paper focuses on the perspective of students and graduates of study programs in sustainable development (“novice” or “learner” perspective). On the other hand, the perspective of internship supervisors in sustainability-oriented companies or in corporate sustainability departments—i.e., professionals from various professional fields who supervise internships—is also taken into account ("expert” perspective).

The results of a 2-year evaluation process of the study programs in sustainable development at the University of Bern from March 2019 to May 2021 serve as a basis for this study. The aim of the present contribution is to gain insights from the results on the assessment of the importance of ESD competencies of students, graduates, and internship supervisors regarding the design of new, as well as the further development of existing sustainability-oriented study programs.

## Materials and methods

### The study programs in sustainable development at the University of Bern and their theoretical foundations

The University of Bern pursues a whole-university approach with respect to the integration of sustainable development in teaching, research, services, and operations [40]. In teaching, a process is ongoing, whereby the integration of sustainable development into all major study programs is taking place. At the same time, the University of Bern also offers special minor programs in respect of sustainable development at the bachelor’s and master's level, which are designed to promote sustainability competencies complementary to the classic disciplinary major programs. A total of four minor programs are offered, namely three programs of 15, 30, and 60 ECTS credits at the bachelor’s level (since 2013) and one master’s program of 30 ECTS credits (since 2015). The latter is designed to be non-consecutive due to the fact that certain disciplinary major programs only allow a minor program either at the bachelor's or only at the master's level, thereby offering students of as many major programs as possible the opportunity to undertake a minor program in respect of sustainable development.

From the beginning, the programs were conceptualized against the background of the competency discussions of the time. These included the general competency discussion [41], the so-called DeSeCo discussion (i.e., Definition and Selection of Competencies) [42–46], the discussion regarding shaping competencies [24–27], and the broader discussion on the competencies in respect of an ESD [16, 17, 20, 28, 29, 33, 37, 47–50]. Equally relevant has been the general ESD discussion, which addresses matters of aligning learning outcomes with intended competency development, appropriate teaching–learning arrangements, topics, content, assessments, and the role of lecturers [4, 51, 52].

In terms of content and topics, the development of the programs was based on the so-called integrative understanding of sustainability as defined by the United Nations [53–56]. Further, in respect of this definition, the UN have already outlined in their report (regarding the World Commission on Environment and Development (WCED) in 1987 [57], which was differentiated in the so-called Rio documents in 1992, as well as in the follow-up conferences), that sustainable development is a transformation encompassing all areas of society. In respect of this understanding—in addition to the general aspects of content (i.e., tackling global challenges as they are concretized, for example, in the Agenda 21 adopted in Rio in 1992, or the Sustainable Development Goals)—the procedural aspects are specified (including in regard to the participation and empowerment of all actors). In order to meet the challenges of sustainable development, actors at all levels of action (individual, local, national, and international) are called upon to act in accordance with the overarching long-term goals of sustainable development.

Accordingly, sustainable development is conceived in the study programs as a comprehensive individual and societal search. Further, it is a learning and shaping process that affects actors in all fields and at all levels of action. In addition, it must also be noted that it consists of a transformation of society, which is referred to by the German Advisory Council on Global Change (WBGU) as a “Great transformation” [58]. The teaching, learning arrangements, and assessments are geared toward the acquisition of competencies and—in addition to the “classic” formats of lectures, exercises, seminars, and individual work—include inter- and transdisciplinary groups; project and research work; case studies; excursions; and practical insights. The possibility to complete an internship, which is assessed with 15 ECTS credits, within the framework of the Bachelor Minor for 60 ECTS credits is particularly worth mentioning (see Sect. 2.3). The lecturers primarily assume the role of learning coaches who support the students in their work and reflection processes.

### The competency framework

In the development of the study programs, the abovementioned discussions at the time regarding “shaping competence” or “Gestaltungskompetenz” [24–27, 52], as well as the competency framework developed by Wiek et al. [20] were crucial (see Sect. 2.1). “Gestaltungskompetenz”, which is mainly discussed in German-speaking countries, means to enable learners to acquire the specific capabilities that are required in order to participate in the elaboration and implementation of solutions regarding the challenges of sustainable development. As such, through this acquisition, they are thus able to participate in the shaping of the future in society. According to de Haan [26], this competency consists of 12 sub-competencies, which are—“namely the ability to:

- gather knowledge in a spirit of openness to the world, integrating new perspectives;
- think and act in a forward-looking manner;
- acquire knowledge and acting in an interdisciplinary manner;
- deal with incomplete and overly complex information;
- cooperate in decision-making processes;
- cope with individual dilemmatic situation of decision-making;
- participate in collective decision-making processes;
- motivate oneself as well as others to become active;
- reflect upon one’s own principles and those of others;
- refer to the idea of equity in decision-making and planning actions;
- plan and act autonomously; and
- show empathy for and solidarity with the disadvantaged.”

In developing their so-called “reference framework for academic program development” and deriving their key competencies in sustainability, Wiek et al. [20] included the relatively generic shaping competency. However, they focused on the creation of a framework for the purposes of revising existing programs, as well as in designing new study programs at the higher education level. The so-called “sustainability research and problem-solving competence” forms the framework competency that enables students to analyze current problem constellations, to create sustainability visions, to elaborate desirable future scenarios, as well as to develop and test strategies for the purposes of problem solving. This framework competency is composed of the following five competencies: “systems-thinking competency”; “normative competency”; “interpersonal competency”; “anticipatory competency”; and “strategic competency”. Brundiers et al. [7] developed this competency model further and created a framework with eight competencies: “systems-thinking competency”; “values-thinking competency”; “futures-thinking competency”; “interpersonal competency”; “intrapersonal competency”; and “implementation competency”. These interact to form the so-called “integrated problem-solving competency”.

During the development of the four minor study programs, it became apparent that, due to the specific circumstances described above, a competency framework could not simply be adopted as a general framework for the programs and that a framework of its own had to be developed. Only when the study programs were concretized in the form of modules, training elements, teaching–learning arrangements and performance assessments did a distinct idea of the competencies to be fostered emerge. From this, the study management compiled an orientation framework with 13 competencies and, for pragmatic reasons, divided these into the four categories “Specialized and methodological competencies” (in German “Fach- und Methodenkompetenzen”); “Personal competencies”; “Social and communicative competencies”; and “Action competencies”.

These categories correspond to what de Haan [26] calls “classical” competency categories, such as “subject and methodological competence”, “social competence”, and “personal competence”. These categories are similarly used by Nölting et al. [59 p. 91], Erpenbeck et al. [41], and Zinn [60]. This “classical” outline was chosen due to the fact that it facilitated communication with lecturers from different disciplines who were not familiar with the discussions regarding ESD at the higher education level, as well as specific ESD competencies at that time. At the same time, this framework allows one to assign aspects of the partly, quite abstract sounding competencies (e.g., systems-thinking competency, strategic competency, etc.) to “classical” competency categories, such as specialized, methodological, and action competencies. The competencies in the four competency categories are detailed as follows (also refer to Table 1):

- *Specialized and methodological competencies:* With respect to an ESD at the higher education level as a minor program complementing a disciplinary major program, it is important that a certain cross-disciplinary sustainability knowledge and cross-disciplinary methodological competencies are present or can be developed and created by means of research [61]. In this context, interconnected, foresighted, and systemic thinking is quite central [7, 20, 29, 33, 62]. Equally important are the specific methodological knowledge of inter- and transdisciplinary procedures and working methods, as well as the ability to access knowledge from outside the discipline and to contribute to inter- and transdisciplinary discourses with one’s own disciplinary knowledge, as well as to work on SD problems [63–65].
- *Personal competencies:* Sustainable development as a process of social negotiation of sustainable solutions, the coordination of the different interests of the various actors, and the resolution of trade-offs requires the ability to recognize and reflect on one's own values and perspective with respect to a sustainable development challenge. This is in addition to empathizing with other perspectives, handling the values and perspectives of other actors, as well as trade-offs and decision-making dilemmas [66]. In the competency model of Brundiers et al. [7], these aspects belong to the values-thinking competency and the interpersonal competency.
- *Social and communicative competencies:* In respect of research and practice, sustainable development requires interdisciplinary and transdisciplinary cooperation regarding certain problems and thus there is a requirement to work in teams and with actors from a wide range of professional fields. Accordingly, it is important to be able to organize work and organizational processes in a multidisciplinary team in a targeted and efficient manner. This should be conducted in order to involve actors from outside academia appropriately in the research processes, as well as to be able to communicate in a comprehensible and target group-oriented manner [67].
- *Action competencies:* It is central that students learn to help shape the transformation [4]. This includes the fact that students engage with real-world challenges and develop contributions in order to address them [19]. In addition, it is required that they are able to contribute their expertise to inter- and transdisciplinary processes [47]. In this regard, it is important to stress that the ability to think and work in an inter- and transdisciplinary manner is considered fundamental in most inter- and transdisciplinary concepts of an ESD [64, 68].

**Table 1:** The 13 competencies in four competency categories that provide the framework for the design of the minor study programs in respect of sustainable development (SD) at the University of Bern

Category	Competency	Explanation
Specialized and methodological competencies	Discipline-independent knowledge of SD (including theories, models, concepts, understandings, and challenges)	In minor programs, which complement disciplinary major programs in fostering ESD competencies, the acquisition of cross-disciplinary knowledge (e.g., regarding SD challenges, transformation concepts, and current research questions) is important
	Methodological expertise, as well as inter- and transdisciplinary approaches and working methods	Appropriate methodological knowledge, especially in regard to specific inter- and transdisciplinary approaches and working methods, is important for the purposes of a cross-disciplinary investigation of SD issues
	Interconnected, foresighted, and thinking approaches in system-dynamic contexts	Networking, foresighted, systemic, or system-dynamic thinking is regarded as a key competency in the context of ESD competency discussions
	Accessing knowledge from other disciplines and using disciplinary knowledge to contribute to inter- and transdisciplinary discourses in order to address SD challenges	In order to deal with challenges that move beyond the major discipline, it is essential to be able to tap into knowledge from outside the discipline and to contribute one's own disciplinary knowledge and skills to interdisciplinary and transdisciplinary discourses in order to address SD issues
Personal competencies	Recognizing and reflecting on one's own perspective on a situation and problem, empathizing with other perspectives, and taking these into account when solving problems	In the context of individual work, as well as in inter- and transdisciplinary collaboration, it is central to recognize and reflect one's own perspective on a situation and problem and to take other perspectives into account appropriately when solving problems
	Handling trade-offs and decision dilemmas	A solution-oriented approach to individual and collective conflicting goals (trade-offs) and decision-making dilemmas is a fundamental challenge for SD
	Handling values, principles, theories, and one's own competencies in a critical and reflexive manner	A reflective and critical approach to individual and societal values, societal norms, guiding principles, theories, and one's own competencies is central to individual and collective solution-oriented action for SD
Social and communicative competencies	Designing work and organizational processes in a multidisciplinary team in a goal-oriented and efficient manner	In research, professional practice, and social engagement, activities for the purposes of sustainable development essentially take place in multidisciplinary teams, which requires that work and organizational processes must be able to be designed in a goal-oriented and efficient manner
	Involving non-academic stakeholders appropriately in the research process	In research, as in scientific professional activity, it is important to adequately involve relevant stakeholders in scientific activity
	Communicating in a comprehensive and target group-oriented manner	In research, professional practice, and social engagement, communication that is comprehensible and appropriate with respect to the target groups in terms of content and form represents an important success factor
Action competencies	Designing, implementing, and reflecting on inter- and transdisciplinary processes on societally relevant topics of SD	In order to fulfil the role of a change agent, it is important to be able to design, implement, and reflect on inter- and transdisciplinary processes in respect of the relevant sustainable development matters
	Working on problems from corresponding professional fields scientifically and making contributions to the further development of the professional fields	With regard to the integration of sustainable development into the professional fields and professional practice, it is central to be able to scientifically address issues of sustainable development in the professional fields and to contribute to the further development of the professional fields
	Participating in the collective work and decision-making processes that are required in order to transform society toward sustainability	In research, professional practice, and social engagement, it is fundamental to appropriately engage in collective work and decision-making processes with one's own sustainability perspective and thus participate in the transformation of society toward sustainability

These competencies should be promoted in all four study programs, but in a level-appropriate manner that corresponds to the scope of the study programs with appropriately concretized learning outcomes, teaching–learning arrangements, content, and assessments.

Thus far, we have not produced a differentiated analysis regarding the question of how comprehensively the framework with the 13 competencies represents the general sustainability competency frameworks mentioned above. Regarding the framework of Brundiers et al. [7], it can be said that the "Systems-thinking Competency", the "Interpersonal Competency", the "Implementation Competency" and the "Integrated Problem-Solving Competency" are relatively well represented. Parts of the "Values-thinking Competency", the "Futures-thinking Competency" and the "Strategic-thinking Competency" are also included in the competency framework, but overall, they are rather in the background when in relation to the other competencies.

### The evaluation of the study programs

For the purposes of the evaluation of the study programs, the faculty responsible for the study programs set up an evaluation commission, which designed the evaluation according to the university guidelines and accompanied it in terms of content and methodology. The evaluation process included: a survey of the students; a survey of the graduates; a survey of the supervisors in the internship companies; two lecturer workshops; a workshop conducted by the students themselves; discussions with the responsible educational partners at other universities (whose students were also admitted to the study programs or to parts of them); two reviews by international experts; and a final workshop by the evaluation commission (which is also responsible for the final report on the evaluation). The answers to the three surveys (in respect of the students, graduates, and internship supervisors) regarding the competencies are included in this analysis, whereby reference is also made to the findings of the other studies when putting the results into context. The three surveys are:

- *Student Survey* (November 2019–January 2020): An email was sent to all 441 students enrolled in one of the four programs during the fall semester 2019. The electronic questionnaire consisted of 67 mostly closed-ended questions. A total of 183 responses were received, of which 124 responses could be included in the analysis, representing a valid response rate of 28.1%. Further, 76% of the 124 respondents identified themselves as female, 23% as male, and one person as other. Moreover, 49 persons studied the bachelor program for 60 ECTS credits, 40 persons in respect of the bachelor program for 30 ECTS credits, and 7 in respect of the program for 15 ECTS credits. In addition, 28 persons studied the master’s program for 30 ECTS credits. At the time of the survey, the students were enrolled in 23 different major programs.
- *Graduates survey* (January and February 2020): An email was sent to all 337 individuals (out of a total of 353) who graduated from a program between 2014 and 2019, and for whom a valid address could be traced. The electronic questionnaire consisted of 59 mostly closed-ended questions. A total of 137 responses were received, of which 121 responses could be included in the analysis, thereby representing a valid response rate of 36%. Just under 74% of the 121 respondents identified themselves as female, whereas just over 26% identified as male, and no person as other. Moreover, 45 persons completed the bachelor program for 60 ECTS credits, 35 persons in respect of the bachelor program for 30 ECTS credits, and 15 regarding the program for 15 ECTS credits. Further, 26 persons completed the master’s program for 30 ECTS credits. The graduates studied a total of 24 major disciplines.
- *Survey of internship supervisors* (January and February 2020): Additionally, by email, 54 (of the total 61) supervisors in the companies were contacted who had supervised at least one student internship in their company from 2014 to 2019 and for whom a valid address could be traced. The electronic questionnaire consisted of 37 mostly closed-ended questions. A total of 21 responses were received, all of which could be included in the analysis, representing a valid response rate of 39%. The internship supervisors were operating in different sectors and could be assigned to public administration, to private and public companies, as well as to non-governmental organizations. The internship supervisors in the companies accompany a student during their 100% 3-month internship (or, correspondingly, longer in the case of reduced employment duration) in an operational unit that is concerned with sustainability issues. During at least half of the internship, the students work on a scientific problem of sustainable development, which is relevant for the company and the professional field. The resulting product is jointly supervised and evaluated by the supervisor in the company and a supervisor at the university. All three questionnaires were pre-tested by several people before mailing. The pre-test served to validate the understanding of the questions, the difficulty of the questions, the sufficient variation of the answers, the continuity of the flow, the interest towards the whole survey and the duration of the survey. Based on the results of the pre-test, the questionnaires were revised.

The questions regarding the importance of the competencies were asked in the same manner to all three surveyed groups, as much as possible. The first question could be asked in the same way to all three groups: "How important do you consider the promotion of the competencies within the study program?" (see Sect. 3.1).

The question on the assessment of the importance of the competencies for the intended professional activity (students' perspective), for the current professional activity (graduates' perspective), and for the professional fields (internship supervisors' perspective) was formulated differently in the questionnaire in each case (see Sect. 3.2):

- Question to students: "How important do you consider the fostering of competencies for your intended professional activity?"
- Question to graduates: "How important do you consider the fostering of competencies for your current professional activity?"
- Question to internship supervisors: "How important do you consider the fostering of competencies within your professional field?"

The third question was addressed only to the graduates: "To what extent do you agree that you have acquired or enhanced the competencies during your SD study program?" (see Sect. 3.3).

Below the respective question, the 13 competencies appeared in random order, and for each competency one could choose from six answers in an opening matrix, starting with "very unimportant"; "rather unimportant"; "partly/partly"; "rather important"; "very important"; and ending with "do not know" (in respect of "completely agree", "rather agree", "partly/partly", "rather disagree", and "do not agree at all"). In the following presentation of the results, the "do not know" answers are omitted and thus only the answers of those who had an opinion are considered. Accordingly, the number of responses per competency and group surveyed varies from competency to competency (as can be seen from the figure texts).

## Results

Section 3.1 presents the results regarding the importance of fostering competencies in the study programs from the perspective of the three groups surveyed, followed in Sect. 3.2 by the results on the importance of the competencies for the intended professional activity (students), for the current professional activity (graduates) and for the professional fields (internship supervisors). In addition, finally in respect of Sect. 3.3, the results on the graduates' assessment of the extent to which they acquired the competencies during their SD studies. In order to identify similarities and differences in the responses of the three groups surveyed, the results are presented comparatively. The results are also sorted according to competency category in order to be able to generate statements regarding the assessment of the importance of the competencies according to a category.

### Importance of fostering competencies in the study programs from the perspective of students, graduates, and internship supervisors

#### Specialized and methodological competencies

**Fig. 1:**
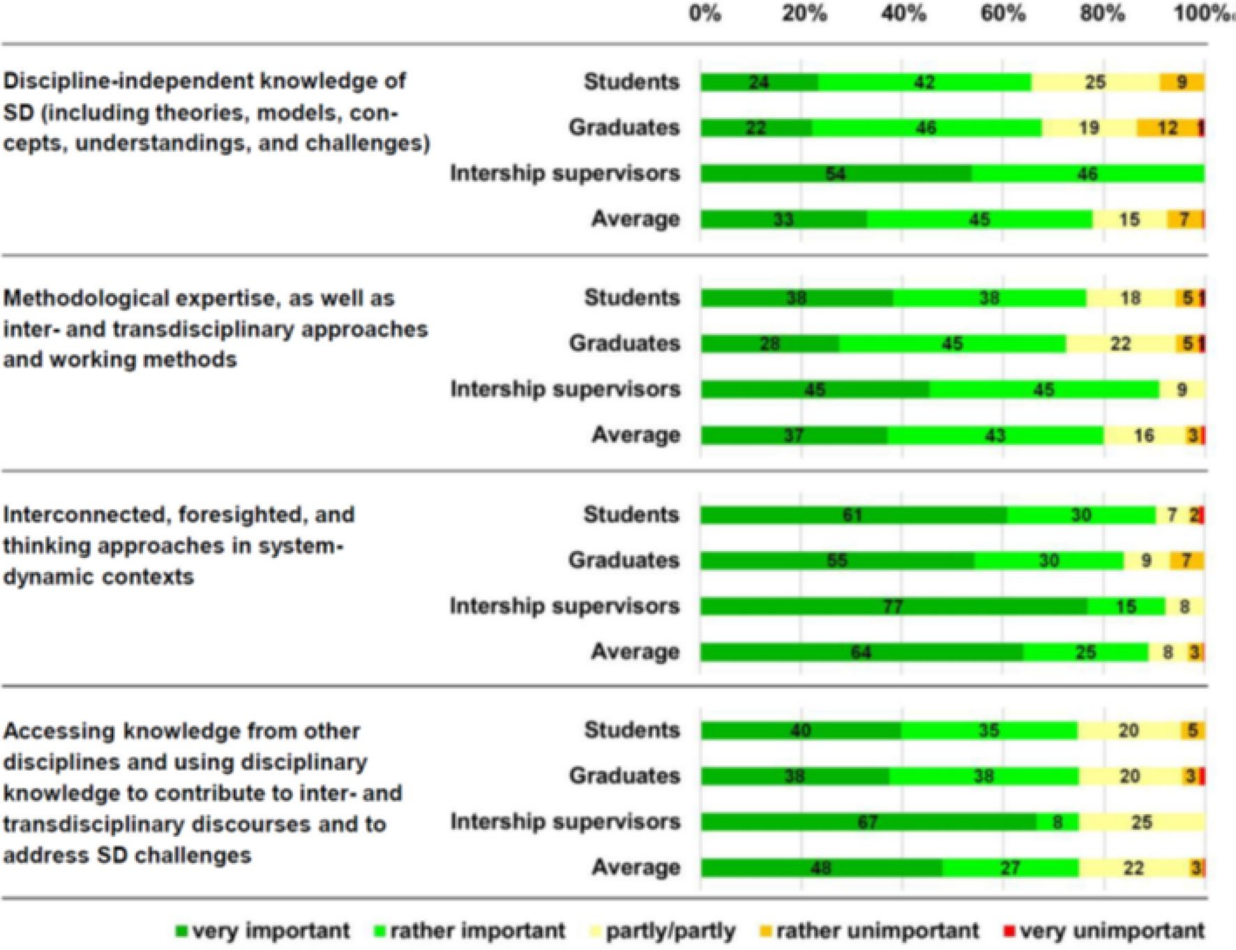
Assessment regarding the importance of fostering specialized and methodological competencies from the perspective of students, graduates, and internship supervisors, as well as the average of the three estimations in % (students are N = 102/102/105/103 from top to bottom; graduates N = 90/87/88/88; and internship supervisors N = 13/11/13/12)

Figure 1 shows that all three groups surveyed considered the four specialized and methodological competencies to be "very important" or "rather important" overall, with average values per competency and group between 66 and 100%. Only very few respondents considered one of the competencies as "rather unimportant" (max. 12%) and only some as "very unimportant" (max. 1.5%).

Across all three groups, the competency "Interconnected, foresighted, and thinking approaches in system-dynamic contexts" was considered by far the most important competency. On average across the three groups surveyed, nearly two-thirds of respondents (64%) considered this competency to be "very important”. In contrast, the competency "Discipline-independent knowledge of sustainable development (including theories, models, concepts, understandings, challenges)" was considered "very important" by an average of only one-third (33%) of the three groups. The two other competencies in this category, "Methodological expertise, as well as inter- and transdisciplinary approaches and working methods" and "Accessing knowledge from other disciplines (…)" are in between with shares of 37% and 48% of the estimations as "very important".

All four specialized and methodological competencies were rated more important by the internship supervisors than by the students and graduates. On average, 61% of the internship supervisors considered specialized and methodological competencies to be "very important", whereas only 41% of the students and 36% of the graduates considered them to be "very important".

#### Personal competencies

**Fig. 2:**
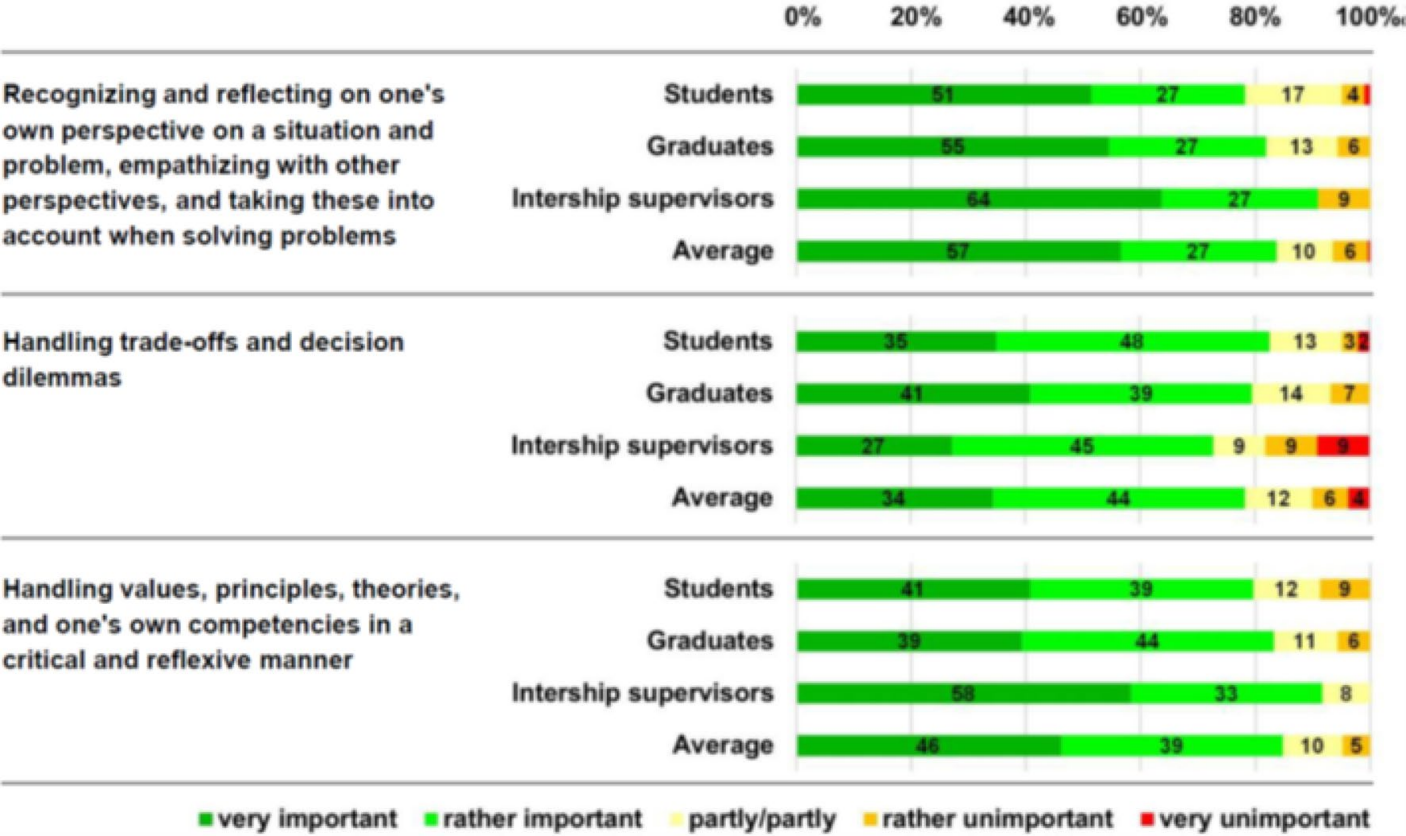
Assessment regarding the importance of fostering personal competencies from the perspective of students, graduates, and internship supervisors, as well as the average of the three estimations in % (students are N = 101/103/103 from top to bottom; graduates N = 88/88/89; and internship supervisors N = 11/11/12)

Figure 2 shows, with respect to all the three groups surveyed, that there is a consideration regarding the three personal competencies as a total (such as the specialized and methodological competencies) to be "very important" or "rather important", with average values per competency and group of between 72 and 91%. Likewise, only a few respondents considered one of the competencies to be "rather unimportant" (max. 9%). Only the competency "Handling trade-offs and decision dilemmas" was considered "very unimportant" by individuals, especially by internship supervisors.

Across all three groups, the competency "Recognizing and reflecting on one's own perspective on a situation and problem (…)" was considered the most important competency. On average, across the three groups surveyed, 57% of respondents considered this competency to be "very important." In contrast, the competency "Handling trade-offs and decision dilemmas" was considered "very important" by an average of just over one-third (34%) of the three groups. The competency "Handling values, principles, theories and one's own competencies in a critical and reflexive manner" is in between with a share of 46% as "very important".

Two of the three personal competencies were rated as more important by the internship supervisors than by the students and graduates, namely the competencies "Recognizing and reflecting on one's own perspective on a situation and problem (…)" and "Handling values, principles, theories, and one's own competencies in a critical and reflexive manner". On average, half of the internship supervisors (50%) considered a personal competency to be "very important"; however it must be noted that this is somewhat lower among graduates (45%) and students (42%).

#### Social and communicative competencies

**Fig. 3:**
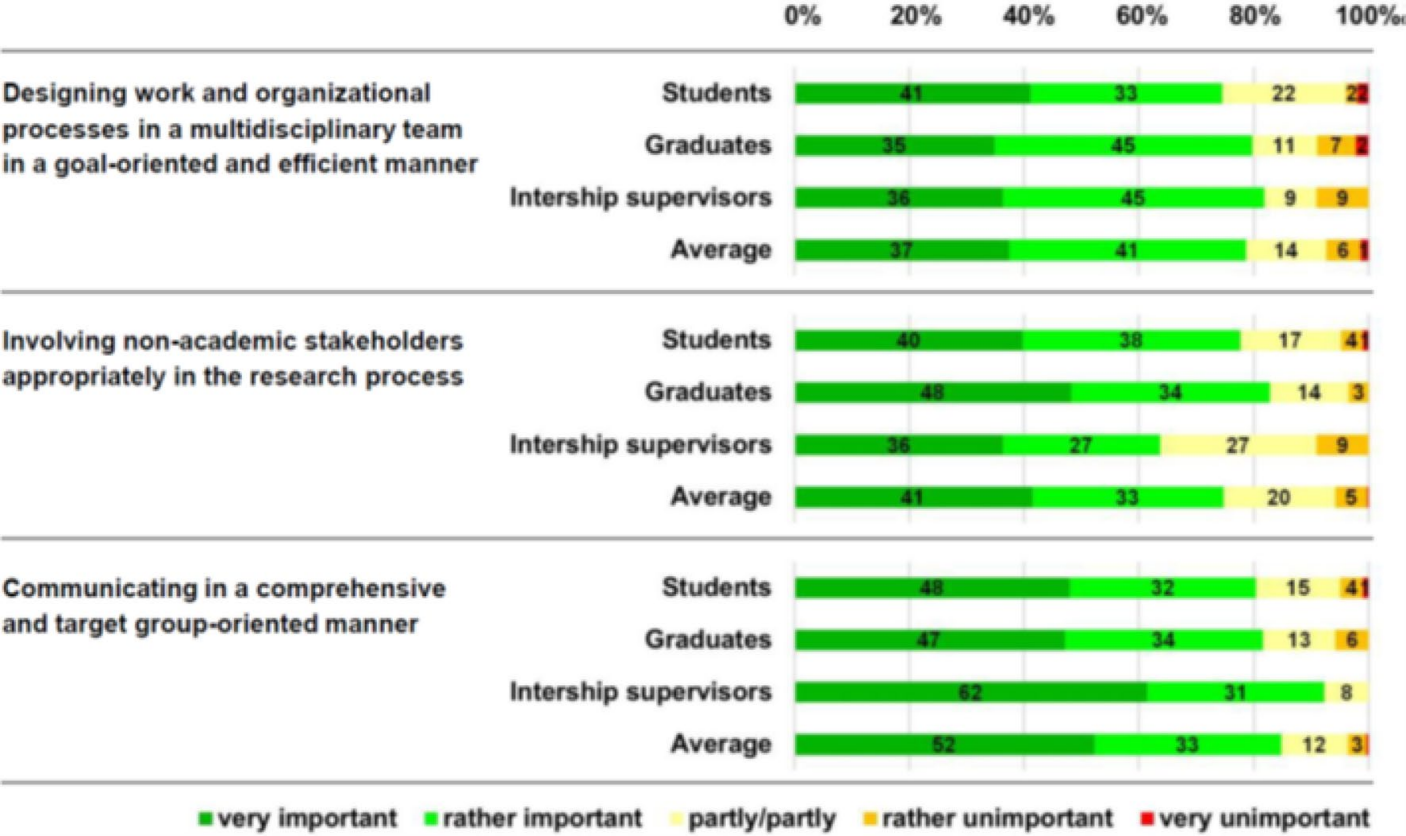
Assessment regarding the importance of fostering social and communicative competencies from the perspective of students, graduates, and internship supervisors, as well as the average of the three estimations in % (students are N = 102/103/102 from top to bottom; graduates N = 89/87/87; and internship supervisors N = 11/13/13)

Figure 3 illustrates that all the three groups surveyed considered the three social and communicative competencies, similar to the competencies in the two categories discussed so far, to be "very important" or "rather important" overall, with average values per competency and group of between 74 and 93%. Likewise, only a small share of the surveyed groups considers one of the competencies to be "rather unimportant" (max. 9%) or "very unimportant" (max. 2%).

Across all three groups, the competency "Communicating in a comprehensive and target group-oriented manner" was considered the most important competency with an average share of 52% "very important". In contrast, the competencies "Designing work and organizational processes in a multidisciplinary team in a goal-oriented and efficient manner" and "Involving non-academic stakeholders appropriately in the research process" were rated somewhat less as "very important" on average across the three groups surveyed, at 37% and 41%, respectively.

In this category, the internship supervisors, with a share of 62% "very important" answers, rate only one of the three competencies as more important than the students and the graduates, namely the competency “Communicating in a comprehensive and target group-oriented manner”. On average, 45% of the internship supervisors considered the three social and communicative competencies to be "very important", whereas 43% of the students and graduates considered these competencies to be "very important".

#### Action competencies

**Fig. 4:**
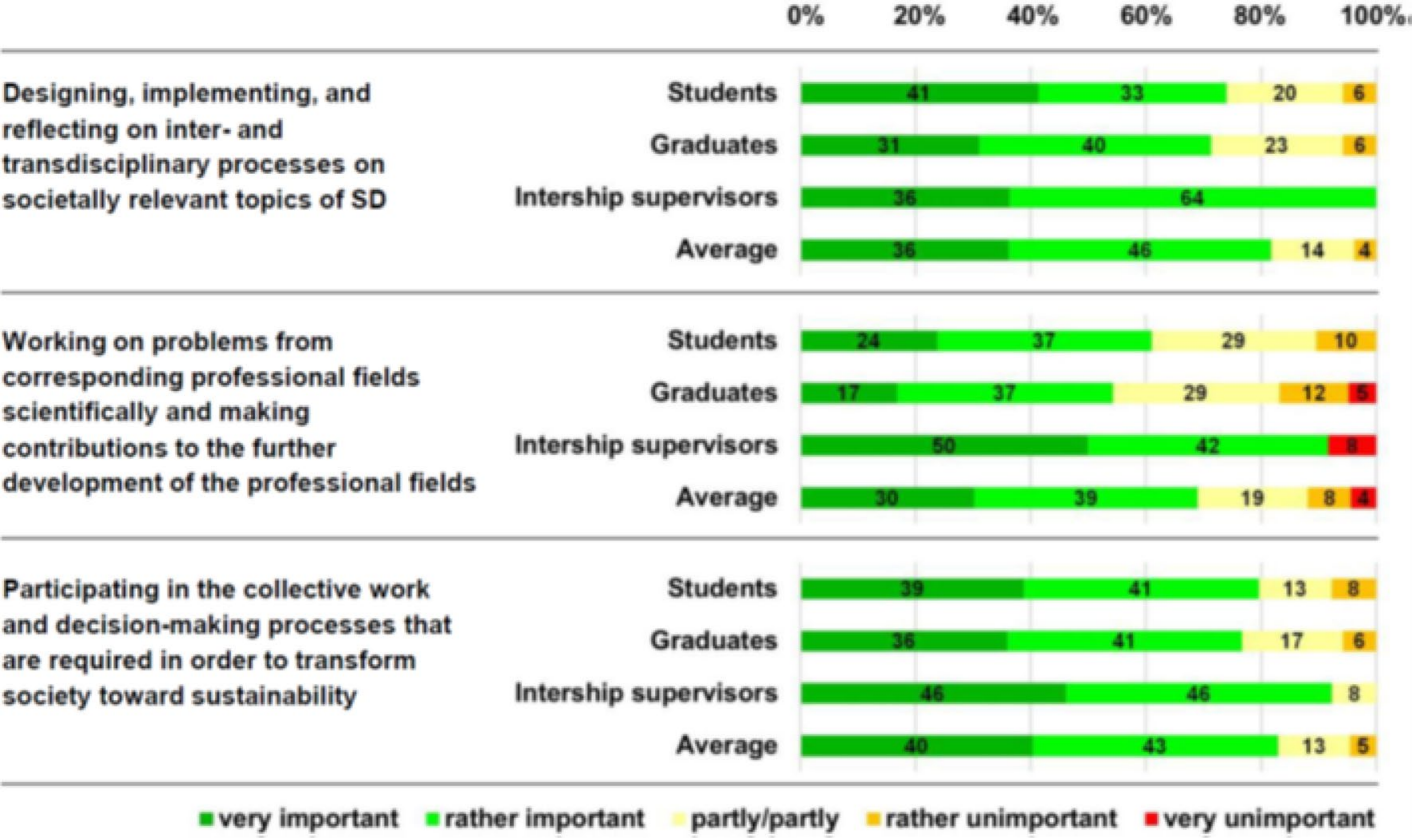
Assessment regarding the importance of fostering in respect of the action competencies from the perspective of students, graduates, and internship supervisors, as well as the average of the three estimations in % (students are N = 104/105/103; graduates N = 87/83/86; and internship supervisors N = 11/12/13)

Figure 4 illustrates that, similar to the competencies in the other three competency categories, the three groups surveyed also considered the three action competencies to be "very important" or "rather important" overall, with average values per competency and group of between 54 and 100%. As with the other three competency categories, only a small share of the groups surveyed considered a competency to be "rather unimportant" or "very unimportant".

Across all three groups surveyed, the competency "Participating in collective work and decision-making processes to transform society toward sustainability" was considered the most important competency within this competency category with a share of "very important" of 40%, ahead of "Designing, implementing, and reflecting on inter- and transdisciplinary processes on societally relevant topics of sustainable development" with 36%.

The competency “Working on problems from corresponding professional fields scientifically and making contributions to the further development of the professional fields” was considered "very important" by an average of only 30% of the three groups, which is the lowest average across all 13 competencies. Regarding this competency, the three groups surveyed also agree the least with respect to its importance. While half of the internship supervisors (50%) rated this competency as “very important”, only 24% of the students, and only 17% of the graduates did so. A similarly striking difference exists only in the estimation of the competency "Discipline-independent knowledge of SD (…)" in the category of specialized and methodological competencies. This competency was rated as "very important" by 54% of the internship supervisors, compared to only 24% of the students and graduates.

#### Overall result

Overall, students and graduates rate a competency as "very important" by an average of 40% and 38%, respectively. The internship supervisors even consider the promotion of a competency during the study program to be "very important" at an average rate of 51%. However, there are differences between the three groups surveyed regarding the estimation of the importance of the competencies:

For the students, the competency "Interconnected, foresighted and thinking in system-dynamic contexts" is by far the most important competency (61% indicated "very important"), followed by the competencies "Recognizing and reflecting on one's own perspective on a situation and problem (…)" (51%) and "Communicating in a comprehensive and target group-oriented manner" (48%). In contrast, only 24% of the students each rate the competency "Designing, implementing, and reflecting on inter- and transdisciplinary processes on societally relevant topics of sustainable development" and the competency "Working on problems from corresponding professional fields scientifically (…)" as "very important", followed by "Handling trade-offs and decision dilemmas" (35%).

The graduates agreed with the students that the two competencies “Interconnected, foresighted and thinking in system-dynamic contexts" and "Recognizing and reflecting on one's own perspective on a situation and problem (…)" represent the two most important competencies. Regarding both competencies, 55% of the respondents indicated that they were "very important". In the third position among the graduates, with 48% of "very important" answers, follows the competency “Involving non-academic stakeholders appropriately in the research process". In addition, followed by the competency "Communicating in a comprehensive and target group-oriented manner" with a share of 47%, which the students rated as the third most important competency. Most students and graduates, therefore, agree on the most important competencies to be promoted in the study program.

Likewise, the graduates agreed with the students that the two competencies "Working on problems from corresponding professional fields scientifically (…)" (only 17% indicated "very important") and "Discipline-independent knowledge of SD (…)" (only 22% indicated "very important") belong to the three competencies that are said to be least "very important". In third place, among the least "very important" competencies, was the competency "Methodological expertise, as well as inter- and transdisciplinary approaches and working methods" with a share of only 28% of "very important" answers.

As for the students and graduates, the two competencies "Interconnected, foresighted and thinking in system-dynamic contexts" (77% indicated "very important") and "Recognizing and reflecting on one's own perspective on a situation and problem (…)" (64%) were among the three most important competencies for the internship supervisors. As the third competency, 67% of the internship supervisors considered the competency "Accessing knowledge from other disciplines (…)" to be very important. In fourth place, with 64% of the "very important" responses, comes the competency "Communicating in a comprehensive and target group-oriented manner", which is one of the three competencies with the highest "very important" percentages among students, but was in fourth place among the graduates.

In contrast, regarding the competencies that are the least "very important", there was only one correspondence between the internship supervisors, the students, and/or the graduates. Similar to the students, the internship supervisors also considered the competency “Handling trade-offs and decision dilemmas” to be less “very important”; whereas, only 27% of the internship supervisors indicated it was "very important".

Considering the average in respect of the estimation regarding the importance of the competencies per competency category, it can be concluded that the students consider the first three competency categories (specialized and methodological competencies, personal competencies, as well as social and communicative competencies) to be roughly equally important—with an average of 41%, 42%, and 43% of the answers being considered as "very important" (Table 2). In contrast, they rated the promotion of competencies in the "action competencies" category as slightly less important, with an average of 35% of "very important" responses.

**Table 2:** Average regarding the assessment of the importance of the competencies per competency category as “very important” (in %)

Competency category	Students	Graduates	Internship supervisors	Average
Specialized and methodological competencies	41	36	61	46
Personal competencies	42	45	50	46
Social and communicative competencies	43	43	45	44
Action competencies	35	28	44	36
Average	40	38	50	43

On average, graduates rated the promotion of competencies in the competency categories as slightly less “very important”, but agreed with the students that the promotion of personal competencies, as well as social and communicative competencies should be considered as more "very important" on average than the competencies in the category of action competencies. On average, graduates considered the promotion of an action competency to be "very important" at only 28%.

Additionally, in respect of the internship supervisors, the competencies in the category of action competencies are, on average, among those that should be least "very important" in the study (on average 44% of "very important" answers). In contrast, 61% of internship supervisors thought that the promotion of specialized and methodological competencies should be considered as "very important" in the study program.

There are only minor differences in the estimation of the importance of fostering the other competency categories. Overall, it cannot be said that one of the three groups surveyed considers a competency category to be significantly more "very important" or significantly less "very important".

#### Preliminary conclusion regarding the estimation of the importance of the fostering of competencies in the study program (all three surveyed groups summarized)

**Fig. 5:**
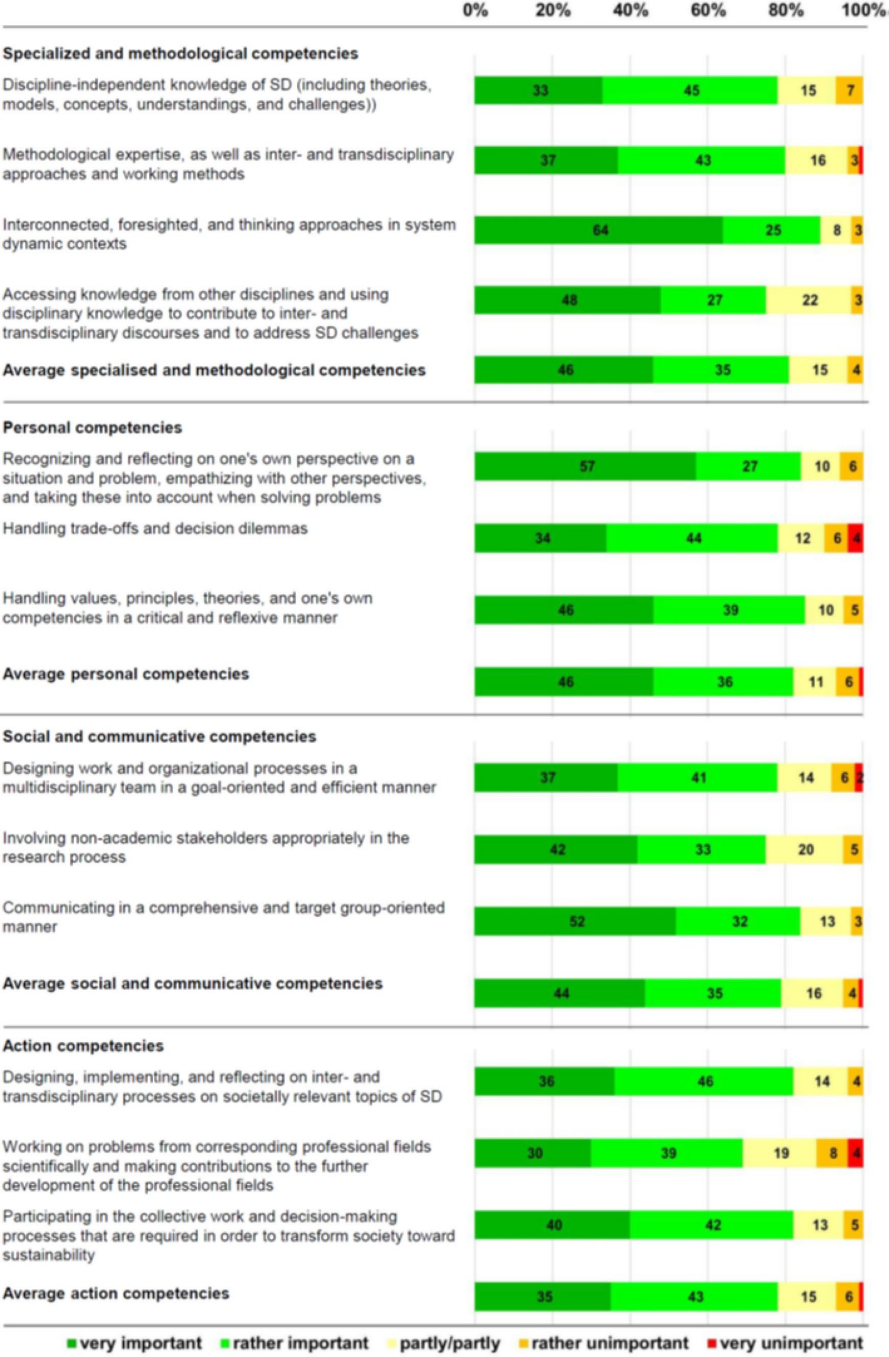
Average regarding assessment of the importance of fostering each competency and competency category in the study programs (average of the three groups surveyed per competency, as well as the average per competency category)

Figure 5 shows the average values in % in respect of the estimations of the importance of fostering the individual competencies, as well as the competency categories across all the three surveyed groups. Based on these averages and the results to date, the following preliminary conclusion can be drawn:

- All competencies were considered important, on average, across the three groups, with average scores ranging from 69 to 89% (i.e., "very important" and "rather important", respectively). On average across the three groups, a maximum of 12% of the respondents considered a competency to be not important (i.e., "rather unimportant" or "very unimportant"). None of the 13 competencies was thus rated as not important.
- Two competencies were considered the most important by all three groups. Accordingly, these also have the highest average values in the estimation as “very important”. These are the competencies “Interconnected, foresighted and thinking in system-dynamic contexts” (64% “very important”) and “Recognizing and reflecting on one’s own perspective on a situation and problem (…)" (57% "very important"). In third place, follows the competency "Communicating in a comprehensive and target group-oriented manner" (52% "very important").
- The competencies "Working on problems from corresponding professional fields scientifically (…)" and "Working on problems from corresponding professional fields scientifically (…)" were considered as the least "very important" competency, on average, across all three groups, with an average of 30% and 33% "very important" responses, respectively. Based on the average values across all three groups, the competency "Handling trade-offs and decision dilemmas" (34% "very important") was among the three least "very important" competencies.
- The average values of the estimations as "very important" per competency category were also higher for the internship supervisors, in all four categories (see Table 2), than for the students and graduates. On average per competency category, internship supervisors rated the competencies within a category as "very important" at 50%, whereas these averages were only at 40% for students and 38% for graduates.
- Based on the average values regarding the estimations of the importance of the competencies of all three surveyed groups and per competency category, the three competency categories “specialized and methodological competencies”, “personal competencies”, and "social and communicative competencies" were considered slightly more important than the category with the "action competencies" (i.e., 37% considered as "very important"), with shares of 46% and 44% "very important" responses, respectively. However, the overall estimations regarding the importance of the various competencies in the competency categories does not allow any statement to be made as to whether a competency category is to be regarded as more or less worthy of fostering by one group of respondents or, on average, by the three groups surveyed.

### Assessment regarding the importance of the competencies for the intended professional activity (students), for the current professional activity (graduates), and for the professional fields (internship supervisors)

#### Specialized and methodological competencies

**Fig. 6:**
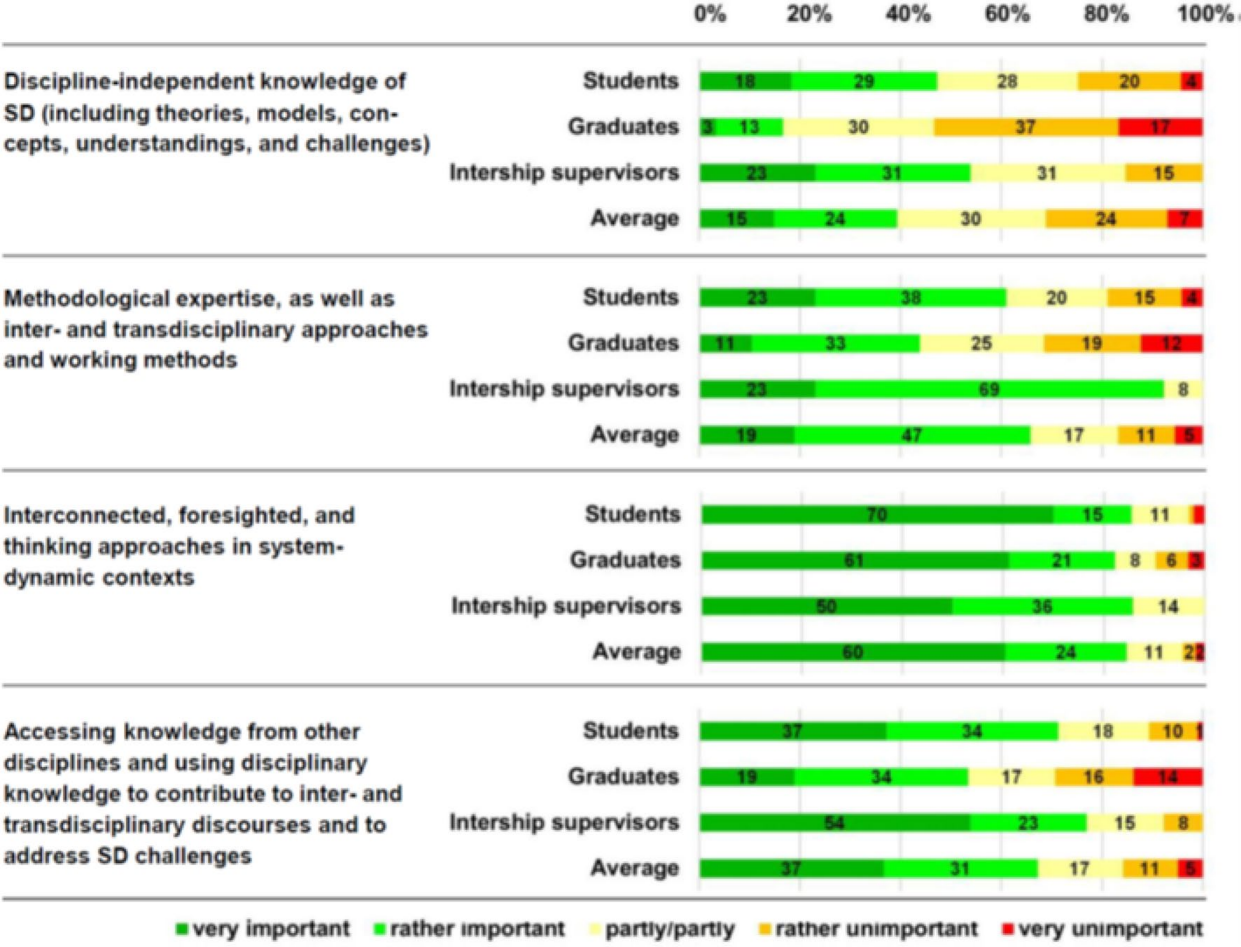
Assessment regarding the importance of specialized and methodological competencies for the intended professional activity (students), for the current professional activity (graduates), and for the professional fields (internship supervisors) in % (students are N = 93/95/97/94; graduates N = 60/57/62/58; and internship supervisors N = 13/13/14/13. In addition, the average of the three estimations is equally weighted)

Figure 6 shows that the estimations regarding the importance of a competency for the intended professional activity (students), the current professional activity (graduates), and for the professional fields (internship supervisors) varied strongly from competency to competency and per surveyed group. Three of the four competencies were rated as "very important" and "rather important" by the majority, but the proportions of "very unimportant", "rather unimportant", and "partly/partly" answers were relatively high, especially in comparison to the estimation of the importance of fostering the same competencies in the study program (see Fig. 1).

The three interviewed groups agreed that the competency “Interconnected, foresighted and thinking in system-dynamic contexts” was the most important of the 13 competencies regarding the intended professional activity (students), the current professional activity (graduates), and the professional field (internship supervisors). In contrast, the competency "Discipline-independent knowledge of SD (…)" was considered as the least important.

The four specialized and methodological competencies were considered, on average, more important by the internship supervisors (for the professional field) and by the students (for the intended professional activity) than by the graduates for their current professional activity.

#### Personal competencies

**Fig. 7:**
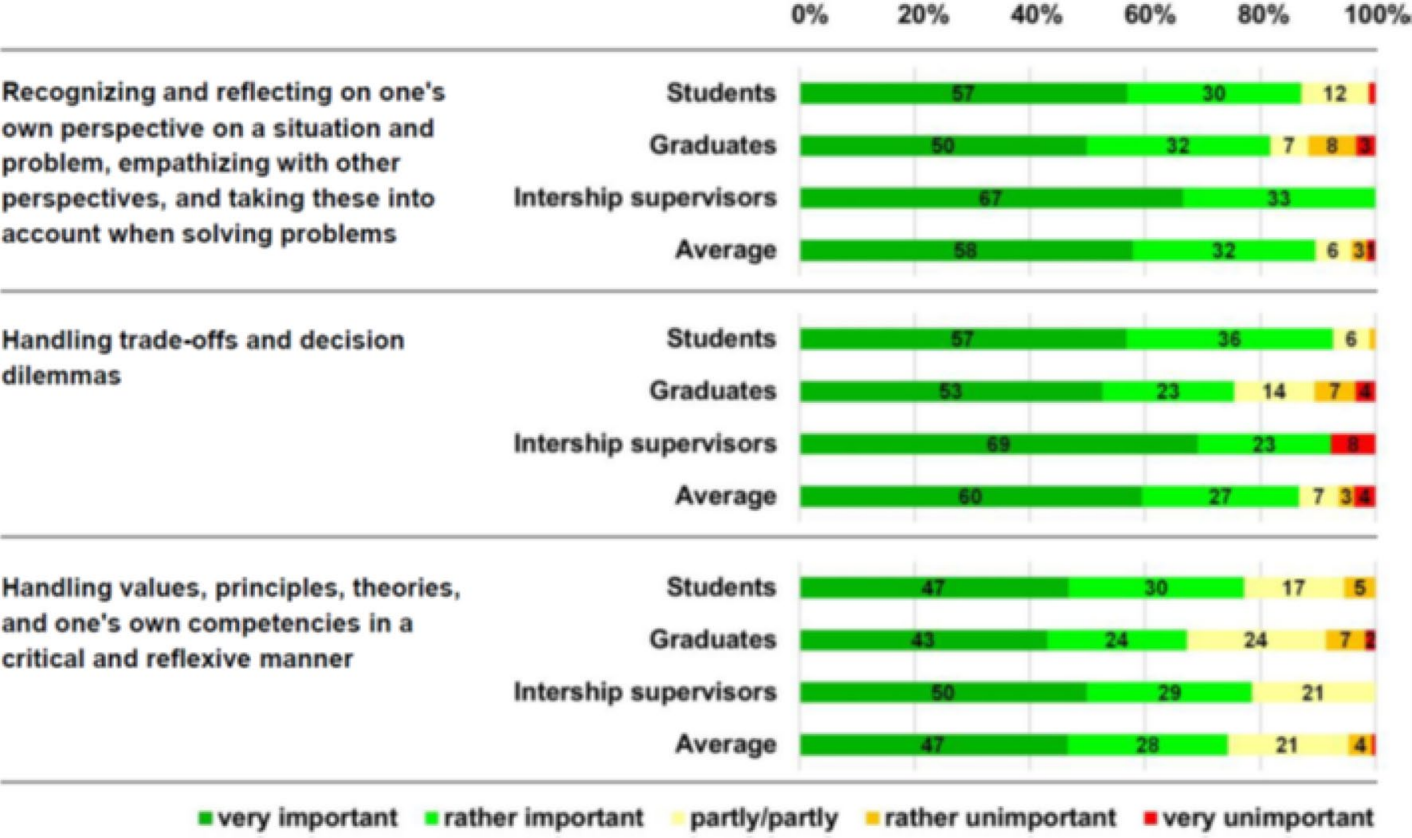
Assessment regarding the importance of personal competencies for the intended professional activity (students), for the current professional activity (graduates), and for the professional fields (internship supervisors) (students are N = 93/95/92; graduates N = 60/57/58; and internship supervisors N = 12/13/14. In addition, the average of the three estimations is equally weighted)

In contrast to the specialized and methodological competencies, all three personal competencies were considered important by all the three groups surveyed (with “very important” and “rather important” responses per competency and group surveyed ranging from 67 to 100%, respectively) (see Fig. 7). The proportions of "very unimportant" and "rather unimportant" responses were relatively low (between 0 and 13% per competency and group), whereas this range extended from 0 to 54% in respect of the estimation regarding the importance of the specialized and methodological competencies.

The differences in respect of the estimation regarding the importance of the three competencies per surveyed group and competency are relatively low. The first two personal competencies were estimated to be somewhat more important than the competency “Handling values, principles, theories, and one's own competencies in a critical and reflexive manner". The internship supervisors estimated the importance of the personal competencies for their professional field with 54% of "very important" answers. This was considered as somewhat more important than the students for their intended professional activity and the graduates for their current professional activity.

#### Social and communicative competencies

**Fig. 8:**
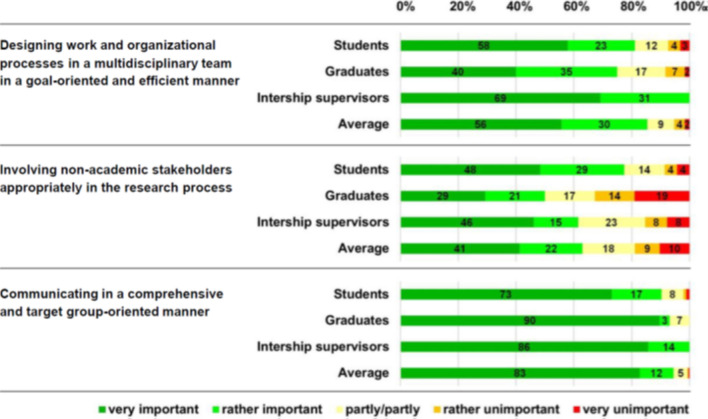
Assessment regarding the importance of social and communicative competencies for the intended professional activity (students), for the current professional activity (graduates), and for the professional fields (internship supervisors) (students are N = 95/93/93; graduates N = 60/58/58; and internship supervisors N = 13/13/14. In addition, the average of the three estimations is equally weighted)

Figure 8 illustrates that all three social and communicative competencies, similar to the personal competencies, were considered as important by the majority (with "very important" and "rather important" responses by competency and surveyed group ranging from 50 to 100%). It is noticeable that the proportions of "very unimportant" and "rather unimportant" answers vary greatly from competency to competency. For example, the accumulated proportion for the competency "Communicating in a comprehensive and target group-oriented manner" per surveyed group was at a maximum of 2%, whereas the same proportion for the competency "Involving non-academic stakeholders appropriately in the research process" per group was up to 33%. This result could have to do with the formulation of the competency, which does not optimally fit the initial question in the questionnaire and required interpretation by the respondents, which is why this result must be put into perspective.

If only the proportions of "very important" responses are considered, it is evident that these are very high (between 73 and 90%) for each group surveyed regarding the estimation of the competency "Communicating in a comprehensive and target group-oriented manner". This competency was considered extremely important by all three groups. The internship supervisors tended to estimate the importance of the three social and communicative competencies higher than the students and graduates.

#### Action competencies

**Fig. 9:**
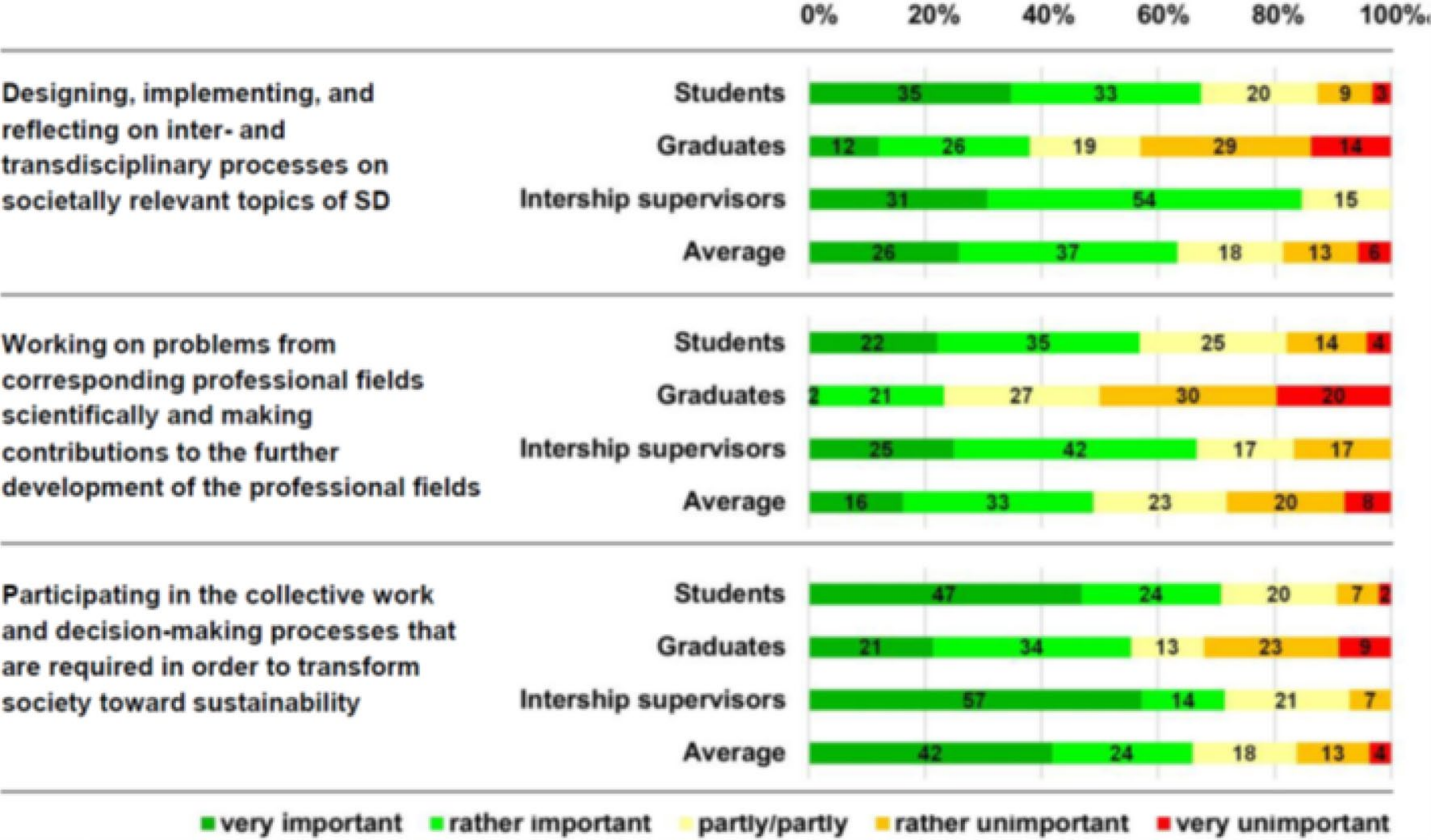
Assessment regarding the importance of the competencies for the intended professional activity (students), for the current professional activity (graduates), and for the professional fields (internship supervisors) (students are N = 95/95/96; graduates N = 58/56/56; and internship supervisors N = 13/12/14. In addition, the average of the three estimations is equally weighted)

Figure 9 shows that the competencies for the intended professional activity (students), for the current professional activity (graduates), and for the professional fields (internship supervisors) were considered important by the majority. However, the values in this respect varied relatively strongly and were markedly lower than those for the estimation regarding the importance of the competencies in the category of personal competencies (see Fig. 7), as well as for two of the three competencies in the category of social and communicative competencies (see Fig. 8).

Based on the "very important" proportions of responses, the competency "Participating in collective work and decision-making processes to transform society toward sustainability" was considered the most important competency. It was noticeable that the graduates considered an action competency for their current professional activity to be "very important" in only 12% of the answers on average. In contrast, both the students and the internship supervisors estimated the three action competencies to be significantly more important. However, these average values were also significantly lower than those in the two categories of social and communicative competencies, and personal competencies.

#### Overall result

On average, 47% of the students estimated the total of 13 competencies as "very important" regarding their intended professional activity (see Table 3). Regarding the internship supervisors, an average of 51% of the competencies were deemed as "very important" for their professional field. However, only an average of 35% of the graduates considered the competencies to be "very important" for their current professional activity, which requires an explanation (see Sect. 4). Nevertheless, there are also major differences between the groups surveyed and from competency to competency that must be taken into consideration.

**Table 3:** Average regarding the assessment of the importance of the competencies with regard to the intended professional activity (students), the importance for the current professional activity (graduates) and for the professional field of the internship supervisors (per competency category as "very important" in %)

Competency category	Students	Graduates	Internship supervisors	Average
Specialized and methodological competencies	37	24	38	33
Personal competencies	54	49	62	55
Social and communicative competencies	60	53	67	60
Action competencies	35	12	38	28
Average	47	35	51	44

In respect of the students, the two competencies "Communicating in a comprehensive and target group-oriented manner" and "Interconnected, foresighted and thinking in system-dynamic contexts" represented, by far, the most important competencies regarding their intended professional activity, followed by the competency "Designing work and organizational processes in a multidisciplinary team in a goal-oriented and efficient manner". In contrast, only 18% of the students estimated the competency "Discipline-independent knowledge of SD (…)" to be very important for their intended professional activity, followed by the competencies "Working on problems from corresponding professional fields scientifically (…)" and "Methodological expertise, as well as inter- and transdisciplinary approaches and working methods".

The two competencies rated by the students as the most important regarding their intended professional activity were also considered by the graduates to be the most important for their current professional activity, namely the competencies "Communicating in a comprehensive and target group-oriented manner" and "Interconnected, foresighted and thinking in system-dynamic contexts". In third place in respect of graduates, follows the competency "Handling trade-offs and decision-making dilemmas" (53%), which for students comes just behind the three most important competencies in fourth place.

This strongly concurring perspective of students and graduates also applies to the least "very important" competencies. In respect of the graduates, the same three competencies were the least "very important" for their current professional activity, which were also the least "very important" for their intended professional activity from the students' perspective, i.e., namely in regard to the competencies "Working on problems from corresponding professional fields scientifically (…)", "Discipline-independent knowledge of SD (…)", and “Methodological expertise, as well as inter- and transdisciplinary approaches and working methods”.

As for the students and the graduates, the same competency is the most important for their professional field in respect of the internship supervisors, namely the competency “Communicating in a comprehensive and target group-oriented manner". Likewise, with very high "very important" shares, two competencies follow, of which one competency each was counted among the three most important competencies by the students and the graduates, which were namely the competencies of "Designing work and organizational processes in a multidisciplinary team in a goal-oriented and efficient manner" and "Handling trade-offs and decision dilemmas".

Even more distinct is the agreement of the estimation regarding the importance of the three least "very important" competencies. The internship supervisors estimated the same three competencies (as the students and graduates) as the least "very important" for their professional field, namely the competencies "Discipline-independent knowledge of SD (…)", "Methodological expertise, as well as inter- and transdisciplinary approaches and working methods", and "Working on problems from corresponding professional fields scientifically (…)".

According to the average estimation regarding the importance of the competencies per competency category, the three groups surveyed agreed on which competencies per category were considered as more important and that which were somewhat less important (see Table 3). The competencies in the social and communication competency category were considered the most important with an average of “very important” percentages of 60% (students), 53% (graduates), and 67% (internship supervisors). All three groups considered competencies in the personal competencies category to be almost equally "very important." The three groups surveyed also agreed that the competencies in the categories of specialized and methodological competencies, as well as action competencies were considered as relatively less "very important". The average values per category and surveyed group in these two competency categories are only half of the average values in the other two competency categories, with 12–38% "very important" shares.

#### Preliminary conclusion regarding the assessment of the importance of competencies for the intended professional activity (students), for the current professional activity (graduates), and for the professional fields (internship supervisors)

**Fig. 10:**
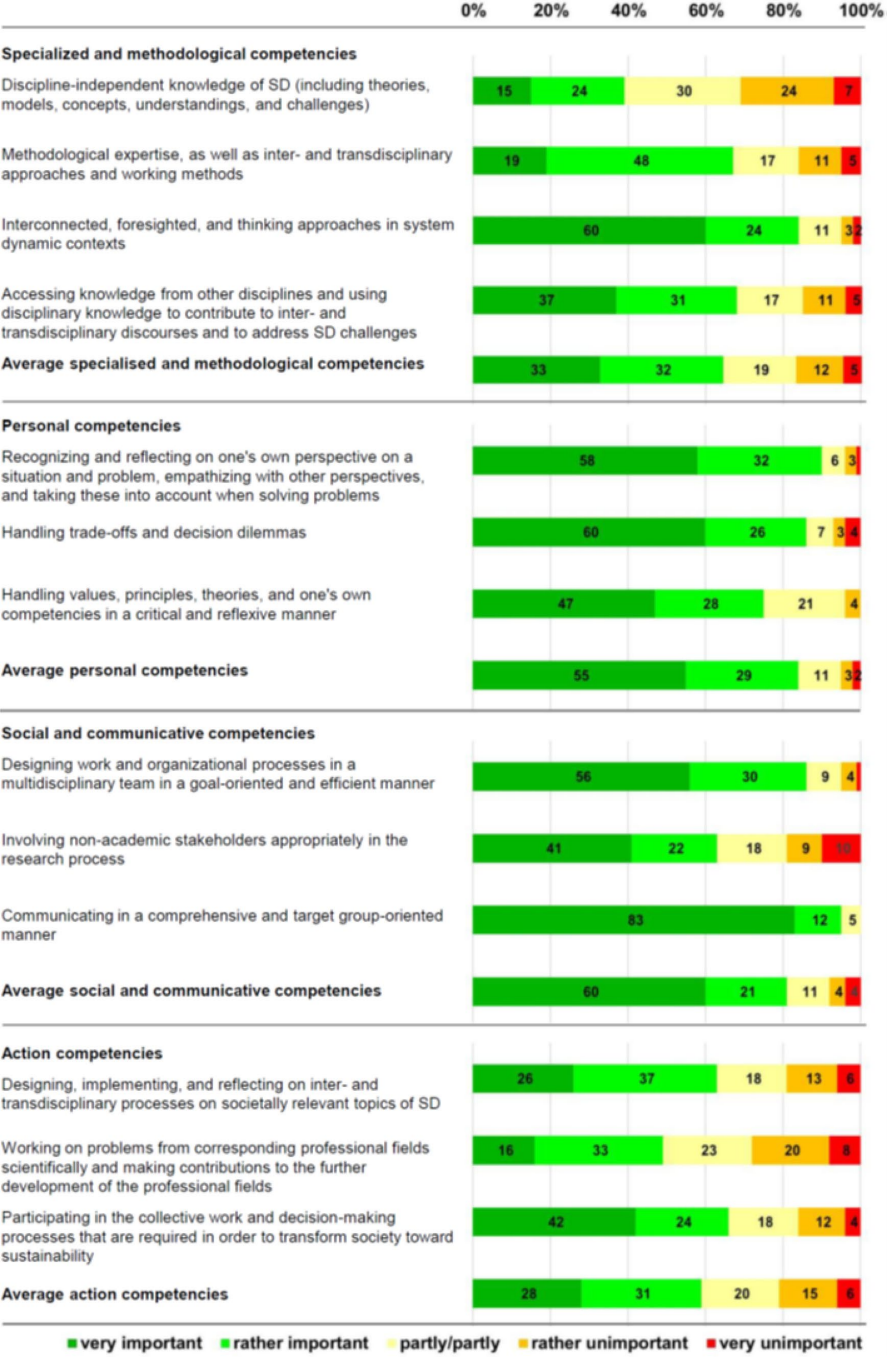
Average regarding the assessment of the importance of the competencies of all three groups surveyed and the categories in % (students: importance for intended professional activity; graduates: importance for current professional activity; and internship supervisors: importance for their professional field)

Figure 10 illustrates what competencies were considered important, on average, across all three groups and how important they were considered to be. Based on these average values and the results described thus far, the following preliminary conclusion can be derived:

On average, the majority of the 13 competencies in the three groups were considered important. On average, no more than 31% of the three groups considered a competency to be unimportant (please note that "rather unimportant" and "very unimportant" are counted together).

- The competency "Communicating in a comprehensive and target group-oriented manner" was considered the most important competency by all three surveyed groups according to the proportions of "very important" responses. Similarly, the two competencies "Recognizing and reflecting on one's own perspective on a situation and problem (…)" and "Interconnected, foresighted and thinking in system-dynamic contexts" were also estimated to be of above-average importance.
- The three groups surveyed agree on the three least "very important" competencies, namely "Discipline-independent knowledge of SD (…)", "Methodological expertise, as well as inter- and transdisciplinary approaches and working methods", and "Working on problems from corresponding professional fields scientifically (…)", although these are not considered "unimportant".
- On average, the internship supervisors considered one competency to be more important than the students and graduates. In respect of 8 of the 13 competencies, the "very important" percentages were higher for internship supervisors than for students and graduates. This includes all three personal competencies.
- On average, the internship supervisors considered the competencies in all four competency categories to be more "very important" than the students and graduates. On average per competency category, internship supervisors estimated the competencies within a category to be "very important" at 51%, whereas this average was only slightly lower for students at 47%. It is noticeable that the graduates estimated the competencies per competency category as "very important" only to an average of 35%.
- In contrast, students, graduates, and internship supervisors agreed on which competencies per competency category were more “very important” and slightly less so in regard to "very important" overall (see Table 3). All the three groups surveyed considered the competencies in the two competency categories "Personal competencies" and "Social and communicative competencies" to be more important than the competencies in the competency categories of specialized and methodological competencies, as well as action competencies. However, based on an overall view of the estimations regarding the importance of the various competencies per competency category, it cannot be stated that a competency category is unimportant from the perspective of a surveyed group (see Figs. 6, 7, 8, 9). Across all three groups, the average cumulative proportions of "very unimportant" and "rather unimportant" estimations were only 5–21%, respectively (see Fig. 10).

### Graduates' estimation of the extent to which they acquired the competencies during their sustainable development study program

**Fig. 11:**
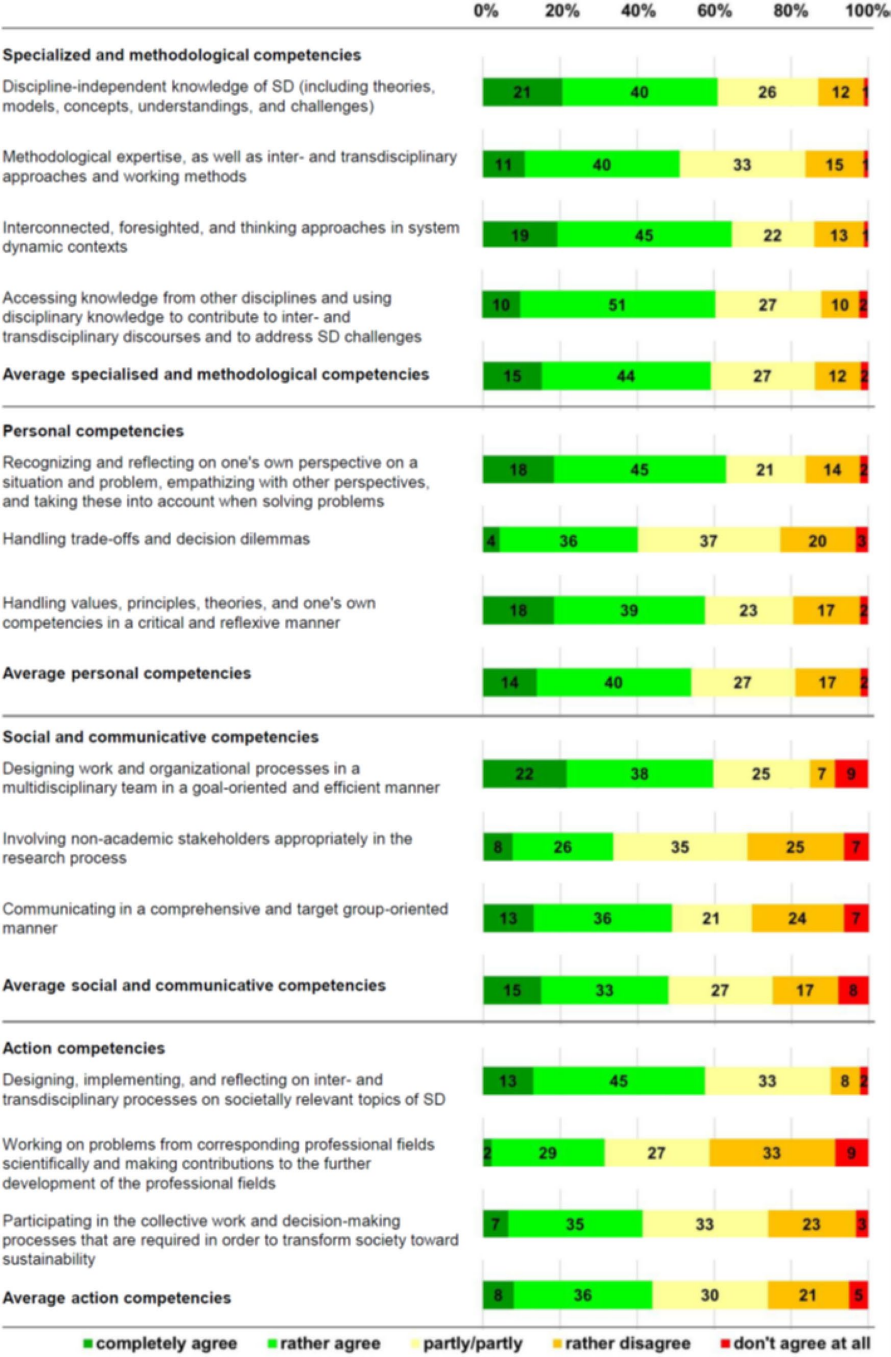
Assessment of the graduates regarding the acquisition and enhancement of competencies during the SD study program (N = 90–92)

Figure 11 shows that, depending on the competency, between 31 and 64% of graduates shared the estimation that they have acquired or enhanced the respective competency. These percentages refer to the total of those who answered "completely agree" or "rather agree" to the above question. The competencies with high scores were distributed across all four competency categories, but it is noticeable that three of the four competencies in the specialized and methodological competencies category are among them. It is also noticeable that the competencies with the lowest values relate to the categories of personal, social, communicative, and action competencies.

However, depending on the competency, only a maximum of around one fifth of respondents indicated "completely agree" (i.e., 2% to a maximum of 22% of respondents). This means that only around one in five graduates is of the opinion that they have acquired or substantially enhanced one of the competencies during the study program.

A relatively high proportion of graduates indicated "partly/partly" (per competency 21–37%). A relatively large number of graduates were, therefore, unable to decide whether they had been able to acquire or enhance the respective competency during the study program or whether they had not been able to acquire or enhance it.

Finally, a relatively large number of graduates (10–42% per competency) were of the opinion that they have not acquired or not enhanced the respective competency at all. More than 30% of the respondents were of the opinion that they have not or not at all acquired or enhanced the competencies "Working on problems from corresponding professional fields scientifically (…)", "Involving non-academic stakeholders appropriately in the research process", and "Communicating in a comprehensive and target group-oriented manner".

If the average values regarding the estimation of the acquisition or enhancement of competencies in a competency category are to be considered, it can be noted that the graduates’ estimate is the highest in respect of the fact that they have acquired or enhanced the competencies in the category of specialized and methodological competencies. Likewise, more than half of the graduates were of the opinion that they have acquired or enhanced the competencies in the category of personal competencies. In contrast, with 48% and 44%, respectively, only just under half of the graduates were of the opinion that they have acquired or enhanced the competencies in the category of social and communicative competencies, as well as in the category of action competencies.

Students, graduates, and internship supervisors were also asked what competencies were missing from the list that should be fostered into the study programs. In addition, the internship supervisors were asked what competencies were missing that are important within their professional field. A total of 18 suggestions were made. The evaluation revealed that the competencies associated with the suggestions were either included in the queried list or were more likely to be classified as learning outcomes subordinate to the competencies. The suggestions related to all four competency categories and confirmed the list with the 13 competencies, whereby the responses of the internship supervisors showed how important they considered the competencies “communicating appropriately and in a target group-oriented manner” and “creative and solution-oriented action” to be for their professional field.

## Discussion

In accordance with the results detailed in Sect. 3.1, most students, graduates, and internship supervisors agreed that all 13 competencies should be fostered in the study programs. In addition, they also agreed that the two competencies "Interconnected, foresighted and thinking in system-dynamic contexts" and "Recognizing and reflecting on one's own perspective on a situation and problem" were the most important. The results regarding the perspective of these three surveyed groups thus confirmed the findings of the surveyed expert groups, namely that the fostering of "systems-thinking competency" in conjunction with the promotion of other competencies, such as "interpersonal competency" and "intrapersonal competency" should be central [7, 20]. Against the background regarding the categorization of the 13 competencies into four categories, the students thus also confirmed the perspective that, in addition to the promotion of specialized and methodological competencies—which is usually the focus in higher education—the promotion of personal, social, communicative, and action competencies was also important. As the 13 competencies, to some extent, cover the competency framework relatively well are called "Integrated problem-solving competency" by Brundiers et al. [7] (see Sect. 2), the results can also be interpreted to mean that the three groups surveyed largely shared the perspective of the experts in respect of promoting a holistic competency development in the study programs that goes beyond the promotion of specialized knowledge and skills. Further, they also agreed that it should include a comprehensive empowerment in order to effectively participate in addressing the societal challenges of sustainable development.

The results in Sect. 3.2 show that the majority of the 13 competencies were also considered important regarding the intended professional activity (students), for the current professional activity (graduates), and for the professional fields (internship supervisors). When compared to the results in Sect. 3.1, however, the estimations of the importance of the competencies differs somewhat. The two competencies that are promoted as the most important in the study program ["Interconnected, foresighted and thinking in system-dynamic contexts" and "Recognizing and reflecting on one's own perspective on a situation and problem (…)"] are, however, also among the five most important competencies for the intended professional activity (students), or current professional activity (graduates), and the professional field (internship supervisors) from the perspective of the three groups surveyed. However, the competencies "Communicating in a comprehensive and target group-oriented manner", "Handling trade-offs and decision-making dilemmas", and “Designing work and organizational processes in a multidisciplinary team in a goal-oriented and efficient manner" were considered equally important or more important.

The three groups surveyed did perceive differences between the importance of a competency in the profession and the importance of fostering it in the study programs. This is reflected in the average estimation regarding the importance of competencies via the competency category. Indeed, while the three groups surveyed considered the importance of promoting competencies per competency category in the study programs to be roughly equally important (see Table 2), the average importance of promoting competencies in the two categories of personal, social, and communicative competencies for the profession was considered to be significantly more important than specialized and methodological, as well as action competencies (see Table 3). This can be interpreted as an indication that the study programs should not simply focus on those competencies that are central to later occupational fields but should also consider, more fundamentally, how the fostering of competencies in the various competency categories should be related in the study programs.

The result regarding the graduates' estimation of the importance of competencies for their current professional activity requires explanation. When compared to the students and the internship supervisors, the graduates estimated a competency for their current professional activity as rather less important than the students regarding their intended professional activity and the internship supervisors in respect of their professional field. A plausible explanation is that the graduates had completed their study program no more than 5 years ago when they were surveyed. Moreover, they are at the beginning of their professional careers and thus they are not yet able to make much use of various competencies in the initial phase of their careers.

As shown in Sect. 3.3, a maximum of roughly one fifth of the graduates (2–22%, depending on the competency) were of the opinion that they have acquired or substantially enhanced a competency during the SD study program. Although an average of just under two-fifths (39%) possessed the opinion that they have acquired or substantially enhanced a competency, a relatively large proportion (10–42%) of graduates (up to two-fifths) possess the opinion that they have not or rather not acquired or enhanced a competency. This cannot be explained by the 12% of graduates who only completed the program with 15 ECTS credits. A plausible explanation is constructed of three parts: namely, that firstly, even in minor programs at 30 and 60 ECTS credits, competencies cannot in actuality be acquired anew or substantially enhanced. Second, too little was communicated to the students during the course of study regarding how the respective educational elements, learning outcomes, and the summative and formative assessment would relate to the development of competencies that is intended to be developed across the study program. Additionally, third, the learning outcomes, the teaching/learning arrangements, and the formative and summative assessment of the various educational elements were not always sufficiently aligned with the competency development.

On the latter point, one result from the two workshops with the lecturers was deemed to be significant: the workshops revealed that a relatively large proportion of the lecturers were not sufficiently aware of the desired competency development across the individual educational elements. This renders it difficult for the lecturers to locate the contribution of the educational elements for which they are responsible to the overall competency development within a study program and to communicate this contribution appropriately to the students. It is also possible that the competency development in all training elements did not take place according to the specifications in the curriculum and in the sense of the formulated learning outcomes.

These aspects point to limitations in respect of the overall methodological setting of this study. First, the evaluation lacked a profound analysis regarding the question whether the different educational elements and their components, such as the formulated learning outcomes, the learning/teaching arrangements, as well as the formative and summative assessments are well aligned with the competency development. Second, the surveys were 'only' self-assessments by students, graduates, and internship supervisors. The addition of other methods, such as an evaluation of student progress assessments throughout the study program or a comparison of an indirect survey of competencies at the beginning of the study program with a survey at the end of the study program, may yield different results. Third, the perspective of the internship supervisors was based on a relatively small sample size (11–14 valid answers per question), which strongly limits the significance of the results in this regard. Fourthly, in respect of the 13 competencies asked, which possess different levels of complexity and that some of them are formulated in a relatively abstract manner, this could have influenced the answers to the questions. Fifth, the internship supervisors did not have the same education as the students and graduates; as such, they may have interpreted various terms included in the formulations of the competencies differently. Additionally, sixth, the collection date of the three surveys was just before the outbreak of COVID-19 in Switzerland. It is possible that the assessment of the competencies would have been different after the outbreak of COVID-19.

The limitations of the methodological setting described above also contain potential starting points for subsequent research. It is desirable to survey the development of competencies across a study program, not only via self-assessments. In addition to the already mentioned possibilities of evaluating assessments and testing competencies at the beginning and at the end of the study program, a systematic observation of competency development by lecturers could also be a possible method. Likewise, the question of which mix of teaching/learning formats and what formative and summative assessments optimally promote the development of competencies across an entire study program should be addressed in greater depth. A further follow-up question is how, in an inter- and transdisciplinary study program in which a multidisciplinary and—at the same time—strong disciplinary team of lecturers teach, the competency development of the students can be ensured throughout the entire study program.

Overall, however, the present results can nevertheless be interpreted to mean that the perspective contained in the frequently discussed competency frameworks, such as those of Wiek et al. [20]; Rieckmann [23, 29]; UNESCO [9]; and Brundiers et al. [7], were conducted, namely, in order to enable students in a competency-oriented and comprehensive manner to take responsibility for meeting the challenges of sustainable development. Furthermore, we can see—through the contributions developed in this study—that this is shared by students, graduates, and internship supervisors.

The experience with the development of the study programs at the University of Bern revealed that these general competency frameworks cannot be directly operationalized. We argue that study program development should consider these existing frameworks but also adapt them to meet context-specific conditions. For example, we can see evidence of this in the form of learning outcomes for modules and the educational elements they contain, which is due to specific conditions (including a multidisciplinary team of lecturers with different ideas). Instead, however, they represent an orientation framework that renders it possible to identify areas of focus and any gaps. The competency framework with 13 competencies that emerged in the context of the development of the study programs at the University of Bern is an example of specifying the general competency frameworks [e.g., 7, 20] for study programs.

## Conclusion

Regarding sustainability-oriented study programs, the following recommendations result from the evaluation of the corresponding programs at the University of Bern. In the new development or further development of sustainability-oriented study programs, it is reasonable to develop a competency framework of one's own and to establish an explicit reference to the broadly discussed competency frameworks. This will enable the identification of any conceptual gaps in the competency-based structure of the programs. In this respect, the competency assessment and the operationalization of the competencies should be considered already during the development of the study program. In the context of this study, we tested the method of scaled self-assessment. However, more innovative instruments of competency assessment are needed that go beyond self-assessment. From our perspective, there is a demand for more studies that address competency assessment to advance research and progress in this area. The lecturers should already be involved in the conception or revision of study programs and the competency structure should be coordinated and communized across the modules and the educational elements (e.g., courses, excursions, projects, assessment of learning outcomes) contained therein—such that a common understanding of the competency structure across the entire study program can emerge in the lecturer team. This should provide a common basis among the lecturers, firstly for the design of the various educational elements of the individual lecturers and, secondly, for the purposes of a coherent communication to the students regarding the contribution of the individual educational elements, teaching/learning arrangements, and assessments in respect of the desired competency development across the individual educational elements.

## Data Availability

The full dataset in an Excel file is available from the corresponding author on request.

## Code availability

The questionnaires for the three surveys are in German and available from the corresponding author on request.
